# Tracking tools of extracellular vesicles for biomedical research

**DOI:** 10.3389/fbioe.2022.943712

**Published:** 2022-11-18

**Authors:** Qisong Liu, Jianghong Huang, Jiang Xia, Yujie Liang, Guangheng Li

**Affiliations:** ^1^ Shenzhen Key Laboratory of Musculoskeletal Tissue Reconstruction and Function Restoration, Department of Orthopaedic Surgery, Shenzhen People’s Hospital (The Second Clinical Medical College of Jinan University), Shenzhen, China; ^2^ Department of Orthopedics, Shenzhen Second People’s Hospital (First Affiliated Hospital of Shenzhen University, Health Science Center), Shenzhen, China; ^3^ Tsinghua University Shenzhen International Graduate School, Shenzhen, China; ^4^ Department of Chemistry, The Chinese University of Hong Kong, Hong Kong, China; ^5^ Department of Child and Adolescent Psychiatry, Shenzhen Kangning Hospital, Shenzhen Mental Health Center, Shenzhen, China; ^6^ Affiliated Hospital of Jining Medical University, Jining Medical University, Jining, China

**Keywords:** exosomes, extracellular vesicles, exosome labeling, exosome tracking, *in vivo* imaging

## Abstract

Imaging of extracellular vesicles (EVs) will facilitate a better understanding of their biological functions and their potential as therapeutics and drug delivery vehicles. In order to clarify EV-mediated cellular communication *in vitro* and to track the bio-distribution of EV *in vivo*, various strategies have been developed to label and image EVs. In this review, we summarized recent advances in the tracking of EVs, demonstrating the methods for labeling and imaging of EVs, in which the labeling methods include direct and indirect labeling and the imaging modalities include fluorescent imaging, bioluminescent imaging, nuclear imaging, and nanoparticle-assisted imaging. These techniques help us better understand the mechanism of uptake, the bio-distribution, and the function of EVs. More importantly, we can evaluate the pharmacokinetic properties of EVs, which will help promote their further clinical application.

## 1 Introduction

Extracellular vesicles (EVs) are lipid-bound vesicles that are secreted to the extracellular space by cells, which contain proteins and nucleic acids in the lumen, and are wrapped with the lipid membrane (Raposo and Stoorvogel, 2013). EVs were discovered decades ago and considered as waste products from cells at first (Johnstone et al., 1987). Only recently, they were found to mediate cell-to-cell communication by transferring molecules from donor cells to recipient cells, thus regulating the signal pathway in target cells (Valadi et al., 2007). Almost all types of cells could release EVs, and they could be detected in all body fluids, such as blood (Deatherage and Cookson, 2012). Moreover, EVs are excellent deliver vehicles for small molecules, proteins, nucleic acids, and nanoparticles, as they are biocompatible, could cross the biological barrier, and protect the inclusions during circulation at the same time (Alvarez-Erviti et al., 2011; El-Andaloussi et al., 2012).

Compared to the bulky studies of applying EVs in therapy development and diagnosis, the study of their behavior in cells and *in vivo* is insufficient due to the lack of related techniques, which should be addressed before the application of EVs as therapeutics (Svenson, 2013; Duan et al., 2021). It is of great challenge to track EVs as they are nanometer-sized and have a complex membrane structure (Mulcahy et al., 2014). Currently, lipophilic dyes are most frequently used for EV staining and fluorescent tracking (Liang et al., 2020a; Xu et al., 2020; Nakazaki et al., 2021). Lipophilic dyes, including PKH26 and DIR, can easily label EVs by intercalating into the lipid membrane. However, these lipophilic dyes form aggregates with similar size as EVs and bring false-positive results (Dehghani et al., 2020). In addition, when tracking with lipophilic-stained EV *in vivo*, the resident cells could also be labeled by the diffused dye (Progatzky et al., 2013). Moreover, the dye has long half-life, so its signal cannot represent EVs in the end (Kim et al., 2019). Therefore, techniques with high efficiency and great tracking capability are in need.

Here, we summarized the arisen tracking methods reported these years in the present study, including fluorescent imaging, bioluminescent imaging, and radio-labeling-assisted imaging, as well as techniques employing nanoparticles for imaging.

## 2 Extracellular vesicles

There are three main types of EVs, namely, exosomes, microvesicles (MVs), and apoptotic body, in which the first two are frequently studied (Todorova et al., 2017). Different EVs have different biogenesis pathways, size, and contents. Exosomes are first generated as intraluminal membrane vesicles in the multivesicular body (MVB) within the endocytic system and then released upon the fusion of the MVB with the plasma membrane (Hessvik and Llorente, 2018). MVs are secreted directly through cell membrane budding (Lv et al., 2019). The size of exosomes ranges from 30–150 nm and MVs from 100–1,000 nm. Large MVs could be isolated by differentiation ultracentrifugation. Small MVs and exosomes are often collected together due to size overlap and the limitation of current purification techniques, which are termed exosomes or extracellular vesicles by different researchers (Théry et al., 2006; Thery et al., 2018). The contents of exosomes are different from the cytosolic contents of the donor cells, and some proteins are sorted into exosomes through the endosomal sorting complex required for transport (ESCRT) machinery (Wei et al., 2021). For MVs, their membrane structure is similar to the plasma membrane of the donor cells, and their inclusions are a portion of cytosol (McDaniel et al., 2013).

**FIGURE 1:**
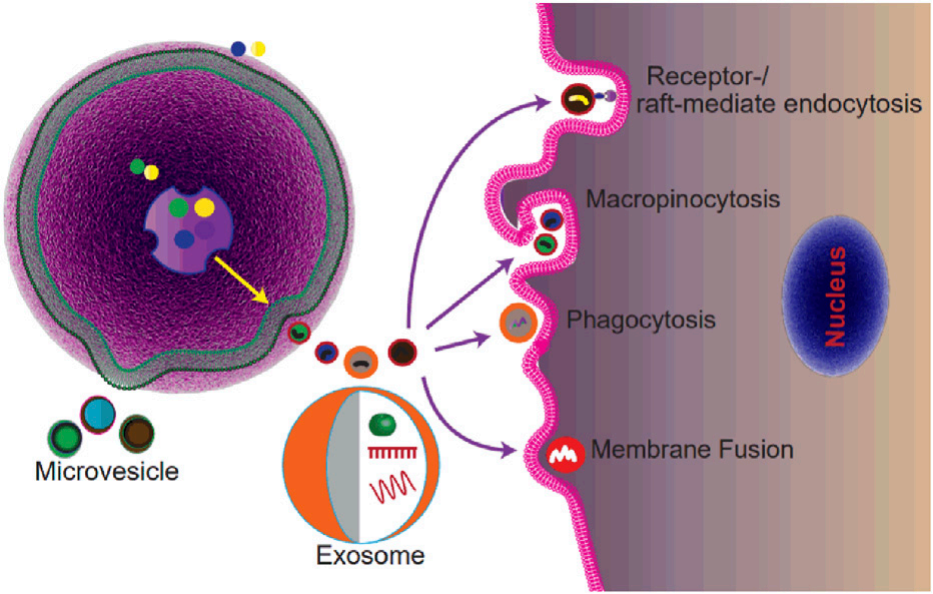
Schematic of the biogenesis and uptake of extracellular vesicles. Exosomes are generated as ILVs within MVBs and secreted after MVBs fusing with the plasma membrane. MVs are generated by the outward budding of the plasma membrane. EVs are internalized by recipient cells *via* micropinocytosis, or phagocytosis, or receptor-/lipid raft-mediated endocytosis, or membrane fusion.

EVs mediate cell-to-cell communication through the transfer of signaling molecules from donor cells to recipient cells, which enables the modulation of various processes in the target cells (Figure 1) (Valadi et al., 2007). Thus, understanding the mechanism of uptake is important as it is the critical step of cell-to-cell communication. However, the clear mechanism of EVs’ uptake is unknown (Mulcahy et al., 2014). Various studies proposed that EV internalization occurs through endocytosis or membrane fusion (Mulcahy et al., 2014). Endocytosis includes a range of uptake pathways including clathrin-dependent endocytosis, caveolin-dependent endocytosis, micropinocytosis, phagocytosis, lipid-raft-involved endocytosis, and receptor-mediated endocytosis (Figure 1). Among these ways, the receptor-mediated endocytosis endows EVs with the ability of selective uptake by specific cell types (Marlin and Springer, 1987; Hoen et al., 2009). It has been reported that EVs with integrin α_6_β_4_ could be specifically internalized by lung fibroblasts, explaining the lung tropism of breast cancer metastasis (Hoshino et al., 2015). In addition to endocytosis, researchers observed that some of the EVs were taken up by target cells through membrane fusion by employing the fluorescent lipid dequenching method (Tian et al., 2013). Overall, EVs could be internalized by virtually all cells tested, and multiple pathways may be involved (Mulcahy et al., 2014). However, for different cell types, the uptake pathways may be different (Mulcahy et al., 2014).

## 3 Labeling of EVs

The efficient labeling of EVs is the essential step for tracking. Labeling methods for EVs include directly labeling EVs and labeling the donor cells in an indirect manner. In the following sections, we summarized the labeling methods from these two categories.

### 3.1 Direct labeling

Direct labeling means directly modifying the isolated EVs, which have membranes to bind to signal molecules and have lumen to encapsulate tracer elements.

#### 3.1.1 Anchoring tracer elements on the EV membrane

To anchor the signal molecules on the EV membrane, the potential binding sites can be the lipid bilayer membrane, the transmembrane protein, and the reactive function group on the membrane.

##### 3.1.1.1 Binding to the lipid bilayer mambrane

The lipid bilayer membrane of EVs facilitates the hydrophobic binding of lipophilic dyes and lipid-tagged molecules and also the electrostatic bonding between the positively charged signal molecules and the negatively charged membrane.

The lipophilic dyes, such as PKH26, can bind to EVs by intercalating into the lipid bilayer membrane, which is the most frequently used method for EV labeling currently (Elmahalaway et al., 2018; Wang et al., 2020; Liu et al., 2021). Moreover, molecules with a lipid tail could also bind to the membrane of EVs due to the hydrophobic binding between the lipid tail and lipid bilayer membrane (Salunkhe et al., 2020). He et al. (2019) synthesized the cholesterol-tagged fluorescent probe complex with two complementary single-stranded nucleic acid chains (A and S). The S chain included the sequence of the aptamer of ATP and had the Cy3 modification at the 3’ end. The A chain included the complementary sequence of the ATP aptamer, a BHQ-1 dye at the 5’ end, and a FAM dye at the 3’ end. They designed this device to monitor the uptake pathway of EVs by cells. When EVs are internalized *via* membrane fusion, the cell membrane showed the green fluorescence of FAM, while if EVs were taken up by endocytosis, the free ATP molecules in the cytoplasm could bind to the aptamer and freed the S chain and showed the red fluorescence of Cy3.

In addition to facilitating the hydrophobic binding, the negatively charged membrane of EVs also allows the attachment of the positively charged dyes by electrostatic bonding (Kim et al., 2016; Feng et al., 2021). Cao et al. (2019) synthesized an aggregation-induced emission luminogen, DPA-SCP, which was positively charged and could attach to the EV membrane for labeling. This method was simple, as it only needed to incubate EVs together with the labeling agents and yet showed superior labeling efficiency.

The labeling methods by anchoring tracer elements to the membrane are often convenient and efficient, which makes their usage frequent. However, the non-covalent binding is not robust and specific, which results in unspecific labeling.

##### 3.1.1.2 Binding to the membrane proteins

Several proteins present on the EV membrane, including EV-specific membrane proteins and some membrane proteins inherent from the donor cells (Hu et al., 2020). Antibodies or aptamers specific to these membrane proteins can be used to EV labeling (Theodoraki et al., 2020; Zhu et al., 2021). Jiang et al. (2018) used gold-carbon dots to label tumor cell-derived EVs by ligating the tumor-specific antibodies to the surface of dots.

The affinity-based binding is tough and specific, but the introduction of antibodies or aptamers may be expensive and laborious. Gao et al. (2018) discovered a peptide, CP05, by phage display, which could bind to EVs specifically. Therefore, EVs could be specifically labeled by dye-tagged CP05. It is well known that the synthesis and modification of peptides are much easier than the production and modification of antibodies or aptamers, and thus this strategy can greatly simplify the labeling.

##### 3.1.1.3 Ligating to the reactive group on EV membrane

There are abundant reactive groups on the surface of EVs, which can react with the tracer molecules (González et al., 2021). However, the reaction should proceed under mild conditions, as EVs cannot tolerate harsh conditions. In this regard, bio-orthogonal reactions are suitable for labeling EVs (Wang et al., 2015). Zhang et al. (2020) employed 4-formylbenzoate to 6-hydrozinonicotinate acetone hydrazine click chemistry to label EVs with quantum dots (QDs) by reacting with the –NH_2_ group on the membrane. This covalent labeling was solid, but it could also react with the free proteins collected together with EVs as current purification methods can not get rid of all the protein contaminates.

#### 3.1.2 Encapsulating the tracer elements in the lumen of EVs

EVs are lipid-bound vesicles. Loading tracers into the lumen can label EVs and protect the tracer elements during circulation at the same time. As EVs are frequently used as delivery vehicles for therapy development, methods used for cargo loading for delivery purpose can be used for labeling as well (Duan et al., 2021).

##### 3.1.2.1 Incubation

Several tracers can be loaded into EVs by a simple procedure of incubation. During incubation, the molecules penetrate the bilayer membrane by physical diffusion or active uptake.

Molavipordanjani et al. (2020) obtained radiolabeled EVs by incubating the fac-[^99m^Tc(CO)_3_(H_2_O)_3_]^+^ synthon with EVs. Then, the bio-distribution of EVs in mouse was imaged using the gamma camera. This labeling process occurs *via* physical diffusion as small molecules can penetrate the membrane spontaneously. Large materials, such as gold nanoparticles, can also be loaded into EVs through incubation. Betzer et al. (2017) reported that glucose-modified nanoparticles could be taken up into EVs *via* an active, energy-dependent manner as EVs could actively uptake glucose.

##### 3.1.2.2 Electroporation

Electroporation is broadly used for cargo loading as the electrical field could generate micro-pores on the membrane, which allows molecules or nanoparticles enter into the vehicle (Xi et al., 2021). Spab et al. (2020) employed electroporation for loading the tracer element, PMA/Au-BSA@Ce6, into EVs and generated the passion fruit-like EV for tracking and treatment at the same time.

##### 3.1.2.3 Sonication

The integrity of the EV membrane could be disrupted by sonication, which allows the co-incubated substances to load into EVs simultaneously upon the reassemble of the membrane (Fu et al., 2020). In addition to the loaded cargo, the processed EVs may have altered potency as the inner contents leak out during sonication, while this outcome may benefit the study under certain circumstances. Jang et al. (2020) utilized electroporation to load the chlorin e6 photosensitizer into tumor cell-derived EVs for photoacoustic imaging and photodynamic therapy. EVs from tumor cells could promote tumor cell proliferation, which might counteract the effects of the photodynamic therapy. However, after sonication, EVs showed no effect on tumor cell proliferation even with a very high dose (50 μg/ml), which possibly resulted from the significantly decreased contents of protein and RNA after sonication.

##### 3.1.2.4 Extrusion

Nanovesicles produced *via* extrusion of cells are another type of attractive delivery vehicles as they have comparable advantages as EVs. Ding et al. (2019) decorated the tracer element, GQDzyme/ABTS, with cell membrane by extruding the erythrocyte, which built a barrier for the nanozyme from circulation, and provided the nanozyme with excellent biocompatibility and stealth ability for long blood circulation. Extrusion of erythrocytes for nanovesicle production shows great potential for therapy development, as erythrocytes are easily obtained from patients and the yield is high (Jo et al., 2014; Kuo et al., 2017).

##### 3.1.2.5 Fusion

Fusion was employed to coat amorphous mesoporous silica nanoparticles with the liposome membrane by Liu et al. (2009) to retain and protect the loaded drugs for further *in vivo* circulation. The fusion occurs spontaneously upon electrostatic bonding between the positively charged silica nanoparticles and negatively charged liposomes. Illes et al. first employed this method to coat the metal–organic framework (MOF) with EVs (Illes et al., 2017; Xhy et al., 2021). The EV coating could seal the MOF from leakage, facilitate the endosomal escape of the drugs, protect them from the circulation, and endow the biocompatibility to the MOF. Compared with other cargo loading methods, the fusion methods retained the membrane integrity to a great extent and thus reserved the advantageous properties of the EV as much as possible.

### 3.2 Indirect labeling

Modifying the donor cells to produce labeled EVs is what called the indirect labeling method. To achieve fine labeling efficiency, the modification should be sorted into the secreted EVs as much as possible.

#### 3.2.1 Genetic engineering

**FIGURE 2:**
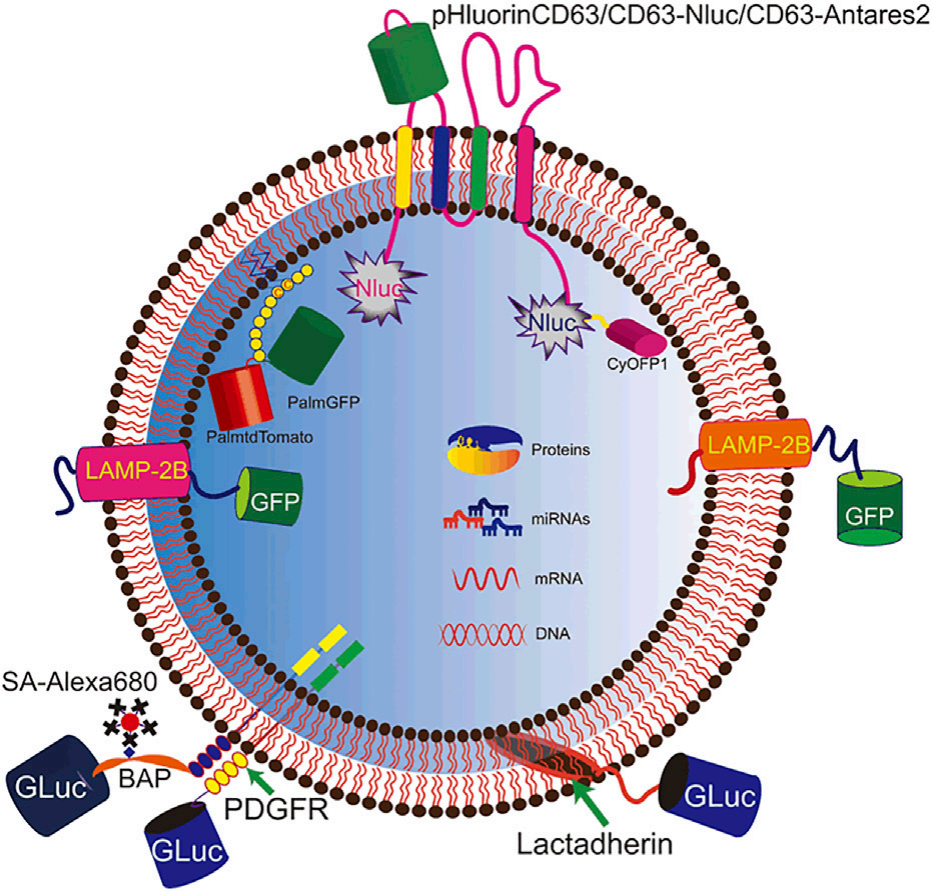
Genetic engineering for EV labeling. Fluorescent and luminescent proteins are tagged to EVs for labeling. Signal molecules (pHluorin, NLuc, Antares2, GFP, and GLuc) are introduced to EVs by tagging to membrane proteins (CD63, Lamp2B, and PDGFR), membrane-binding protein (lactadherin), and lipid tail (PalmGFP).

Genetic engineering is the most frequently used method for EV labeling. The tracer elements were expressed either as recombinant proteins on the EV membrane or as cargo in the EV lumen (Figure 2). CD63, the exosomal marker protein, is often edited for labeling (Logozzi et al., 2009). Fluorescent or luminescent tags are engineered either to the terminus or to the extracellular loop of CD63, which are tagged either inside or outside of EVs, respectively, such as pHluorin, NLuc, and Antares2 (Sung et al., 2015; Hikita et al., 2018; Verweij et al., 2018; Hikita et al., 2020). The M-shaped topology of CD63 possesses restrictions for engineering as both the N- and C-terminus are distributed inside the vesicles. Curley et al. (2020) designed several CD63 truncates for tagging and found a distinct sequence of CD63 for flexible tagging. In addition to CD63, other exosomal proteins employed for tagging tracer proteins are Lamp-2B, lactadherin, and PDGFR (Alvarez-Erviti et al., 2011; Ohno et al., 2013; Takahashi et al., 2013; Liang et al., 2020b; Liang et al., 2021). However, engineering a single protein for labeling may result in bias as it would only label one subtype of EVs. Gupta et al. (2021) systematically screened the exosomal proteins for loading and found that the combination of two exosomal proteins greatly increases the efficiency for therapy development.

In addition to tagging to the exosomal proteins, fluorescent proteins can also be engineered to express in the lumen of EVs. Lu et al. engineered the donor cells to express PalmGFP, which could attach to the EV membrane through the lipid tail, and thus increased the exosomal loading of GFP (Lai et al., 2015; Lu et al., 2021). Other than fluorescent or luminescent elements, nucleic acids can be used as tracer elements as well. Bala et al. (2015) introduced miR-155 to the lumen of EVs through engineering the donor cells, and then they analyzed the bio-distribution of EVs in mouse *via* quantitative PCR because of the absence of miR-155 in mouse cells. Very recently, Ferreira et al. (2022) found that proteins containing the KFERQ motif pentapeptide could be loaded into EVs *via* the membrane protein Lamp2A. The recombinant protein of mCherry tagged with the KFERQ-like sequences was enriched in EVs, which provided a powerful method for EV labeling.

#### 3.2.2 Metabolic labeling

During cell metabolism, cells uptake nutrition from the environment, such as H_2_O and glucose. The cells will be metabolically labeled when cultured in a medium with labeled nutrient substances and produce labeled EVs. Horgan et al. (2020) obtained deuterium-labeled EVs by culturing cells in a medium containing deuterium oxide, deuterated choline chloride, or deuterated d-glucose. The labeled EVs acted as a bio-orthogonal Raman-active tag for direct Raman identification of EVs during cell internalization. Lee et al. (2018) also metabolically incorporated the active function group, -azido, into EVs by incubating the donor cell with azido-sugar. Then, EVs were further modified by click chemistry. This type of labeling methods provides a simple and convenient manner for introducing tags to EVs, which has a minimum impact on the properties of the produced EVs.

#### 3.2.3 Lipid exchange

Since MVs are generated from direct membrane budding, they have similar membrane structure as the parental cells (Lv et al., 2019). Introducing tags to the cell membrane of the donor cells can produce tagged MVs spontaneously. Chen et al. incubated the donor cells with biotin-functionalized phosphatidylethanolamine to produce biotin-labeled MVs (Valadi et al., 2007; Chen et al., 2015). These MVs were further labeled with streptavidin-functionalized quantum dots (QDs) for imaging (Valadi et al., 2007; Chen et al., 2015).

#### 3.2.4 Labeling donor cells

Stained cells can also produce stained EVs. Monopoli et al. (2018) used an amphiphilic dye to stain the donor cells. The cells could produce NIR-labeled EVs for imaging. The labeling processes included steps of uptake of dyes by cells first and second the secretion of labeled-EVs, which was a route similar to the exocytosis of the anti-cancer drugs from tumor cells as a drug-resistance defense of the cells.

## 4 Imaging methods

Several imaging modalities are employed for EV imaging till now. We categorize them into four types: fluorescent imaging, bioluminescent imaging, nuclear imaging, and nanoparticle-based imaging. Table 1 contains the list of the advantages and disadvantages of the principal imaging approaches.

**TABLE 1:** Comparison of the principal imaging methods for EVs.

Imaging method	Examples of tracer element	Pros	Cons	Reference
Fluorescent imaging	DiR, Cy5.5, enhanced GFP, AIEgens, and pHluorin	Simple labeling procedure, high labeling efficiency, imagers are widely available, medium sensitivity, and medium signal-to-noise ratio	Low spatial resolution, low penetrability, and non-specific labeling in case of lipophilic dyes	Lai et al. (2015); Lee et al. (2018); Cao et al. (2019); Sun et al. (2019); Verweij et al. (2019)
Bioluminescent imaging	RLuc, Gaussian luciferase, NanoLuc, and ThermoLuc	High to medium labeling efficiency in case of different approaches, specific labeling, imagers are widely available, high sensitivity, and high signal-to noise ratio	Genetic labeling, substrate needed, low spatial resolution, and low deep tissue penetration	Gangadaran et al. (2017); Gupta et al. (2020)
Nuclear imaging	^99m^Tc, ^111^Indium, and ^64^Cu	High labeling efficiency, high sensitivity, excellent signal-to-noise ratio, and high deep-tissue penetration	Hazardous, low spatial resolution, specialized infrastructure needed, and high cost	Molavipordanjani et al. (2020); Gangadaran et al., (2017); Faruqu et al. (2019); Banerjee et al. (2019)
MRI imaging	^64^Cu	High deep-tissue penetration and high spatial resolution	Low sensitivity and high cost	Banerjee et al. (2019)

### 4.1 Fluorescent imaging

Fluorescent imaging is a strategy extensively used in molecular and cellular biology (Lu et al., 2019).Fluorescent microscopy is easy to operate and could provide information in real time and noninvasively. Chemical dyes, fluorescent proteins, and aggregation-induced emission luminogens (AIEgens) are well-used indicators for fluorescent imaging. However, there are several disadvantages for *in vivo* fluorescent imaging, as the fluorescent signal has low penetration capability and high background due to the auto-fluorescence of the tissue, which makes it more suitable for *in vitro* imaging (Ntziachristos et al., 2003). Plenty of studies tracked labeled EVs *via* fluorescent imaging, in which the signal could keep constant or altered upon environment change. Representative examples are listed in Table 2.

**TABLE 2:** Representative examples for labeling and *in vivo* tracking of EVs.

Labeling agent	EV source	Imaging time-point	Imaging technique	Admin. route	Reference
**DiR**	Tissue explant	2 h	IVIS spectrum confocal microscopy	i.v.	Sun et al. (2019)
					Lee et al. (2018)
**Cy5.5**	Breast cancer cells (MDA-MB-231 and MCF7 cells)	4 h and 24 h	IVIS spectrum	i.v.	
**Enhanced GFP and tandem dimer Tomato**	Xenograft generated with PalmGFP-transfected EL4 cells (mouse thymoma cell line)	9 days	Multiphoton intravital microscopy	Endogenous-generated EVs	Lai et al. (2015)
**AIEgens (DPA-SCP)**	Human placenta-derived MSCs	1 h, 1 day, 3 days, 5 days, and 7 days	IVIS spectrum confocal microscopy	i.v.	Cao et al. (2019)
					
**pHluorin**	Yolk syncytial layer of zebrafish	Zebrafish embryos 3 days post-fertilization	Electron microscopy and fluorescent microscopy	Endogenous-generated exosomes	Verweij et al. (2019)
**Cre-loxP system with CFP, RFP, and GFP**	Transplanted MDA-MB-231 cells generated highly metastatic mammary tumors	\	Multi-photon high-resolution intravital imaging and confocal microscopy	Endogenous-generated exosomes	Zomer et al. (2016)
**CRISPR-Cas9 system with tdTomato**	Tumor xenograft generated with transfected melanoma cells	21 days	Confocal microscopy	Endogenous-generated exosomes	Ye et al. (2022)
**RLuc**	CAL-62 (thyroid cancer cells) and MDA-MB-231 cells	10 min, 30 min, 1 day, 2 days, 3 days, 6 days, 9 days, and 12 days	IVIS spectrum	i.v.	Gangadaran et al. (2017)
**ThermoLuc**	HEK-293T cells	30 s, 60 s, 90 s, 120 s, and 150 s	IVIS spectrum	i.v.	Gupta et al. (2020)
^**99m**^ **Tc**	HEK-293T transfected with HER2 target motif on the Lamp2B protein	1 h and 4 h	Gamma camera	i.v.	Molavipordanjani et al. (2020)
^**99m**^ **Tc**	Red blood cells	1 h and 3 h	Gamma camera	i.v.	Gangadaran et al. (2017)
^**111**^ **Indium**	Melanoma cells (B16F10)	0, 4 h and 24 h	Gamma camera	i.v.	Faruqu et al. (2019)
^**64**^ **Cu**	hUCB-MNCs	1 h, 1.5 h, 2 h, and 3 h	Gamma camera and MRI scan	i.v.	Banerjee et al. (2019)
**Gold nanoparticles**	Human MSCs	24 h	Micro-CT imaging	i.v. and IN	Betzer et al. (2017)
**Quantum dots**	HUVEC cells	24 h	IVIS spectrum	i.t.	Chen et al. (2015)
**Chlorin e6 photosensitizer**	MIA-PaCa-2 cells	6 h	IVIS spectrum	i.v.	Jang et al. (2020)

IVIS, *in vivo* imaging system; MSCs, mesenchymal stem cells; i.v., intravenous injection; IN, intranasal administration; i.t., intra-tumor injection.

#### 4.1.1 With constant fluorescent

The tracking of the constant signals can reveal the distribution of labeled EVs in cells or *in vivo*. For this purpose, chemical dyes, fluorescent proteins, and AIEgens with the conventional design can provide satisfactory results.

Chemical dyes are widely used for tracking EVs as they have stable fluorescent signals, minimal impact on EV property, and simple labeling procedures. Sun et al. (2019) employed the lipophilic dye, DIR, for EV tracking. They analyzed the bio-distribution of the labeled EVs using the confocal laser scanning microscope (CLSM) with the tissue sections. By comparing the bio-distribution of EVs with or without ultrasound-targeted microbubble destruction (UTMD), they concluded that UTMD provided an excellent strategy for the targeted delivery of EVs. Lipophilic dyes are convenient and efficient for EV labeling. However, aggregate formation, possible staining of resident cells, and long half-life limit their tracking efficiency (Dehghani et al., 2019). To achieve better performance, the purification step for the stained EVs should be cautiously performed. Pužar Dominkuš et al. (2018) reported that only 10% of particles collected by ultracentrifugation after staining with lipophilic dyes were labeled EVs, and they suggested to use the sucrose gradient to improve the purity. In addition to lipophilic dyes, cyanine dyes are broadly used for labeling in cell research studies (Shi et al., 2016). Usually, they label biomacromolecules *via* covalent ligation, and thus several functionalized derivatives are commercially available for this purpose. Lee et al. (2018) labeled EVs with Cy5.5 *via* click chemistry-assisted ligation and tracked the labeled EVs *in vivo* by CLSM with the tissue sections. They found that EVs derived from highly metastatic cancer cells had selective organ distribution. Chemical dyes are stable, easy to use, and sensitive, but they have limited penetration depth, which hinders the application in deep body structure imaging.

Fluorescent protein is another well-used imaging indicator for cell research. Lai et al. (2015) labeled EVs with PalmGFP to monitor the communication among tumor cells and between tumors cells and the environment *via* EV exchange by multiphoton intravital microscopy. They found that EVs released by tumor cells distributed intracellularly, or associated with the cytoplasmic membrane, or outside of the tumor cells. They also had the time-lapse recordings, which showed that the EVs could transport individually in small, fast-moving clusters. This indicated that EVs from tumor cells could be internalized by surrounding cells, such as the tumor-infiltrating immune cells.

Another popular fluorescent indicator, AIEgens, is luminogens with aggregation-induced emission properties (Huang et al., 2020). AIEgens show excellent results for biomedical imaging with the advantages of excellent biocompatibility, super resistance to photobleaching, and high signal-to-noise ratios. Cao et al. (2019) employed the positively charged AIEgens, DPA-SCP, for labeling EVs through electrostatic bonding, which required only a simple labeling procedure but exerted superior labeling efficiency. Furthermore, they found that DPA-SCP could track EVs *in vivo* noninvasively, precisely, and quantitatively without impacting their therapeutic effects. So DPA-SCP supported the convenient, precise, noninvasive, and real-time tracking of EVs, which was superior over other fluorescent dyes.

#### 4.1.2 With changeable fluorescent signal

Tracking with the changeable fluorescent signal can provide more information than tracking with the constant fluorescence signal, such as EV secretion and cellular uptake. Moreover, it can be used to monitor the journey and fate of endogenous EVs, but not pre-isolated EVs, which reveals the *in situ* communication between cells *via* EV exchange.

pHluorin is a pH-sensitive green fluorescent protein and represents a powerful tool for monitoring EV secretion and the fusion of MVB with the plasma membrane (Sung et al., 2015; Bebelman et al., 2020; Sung et al., 2020). Verweij et al. (2019) applied the CD63-pHluorin-engineered zebrafish embryos for live-tracking of the biogenesis, journey, and internalization of individual endogenous EVs *in vivo*. They found that EVs released from the yolk syncytial layer were first captured, endocytosed, and degraded by the patrolling macrophages and endothelial cells during the blood circulation. The CD63-pHluorin zebrafish embryos represented a powerful tool to study the function of endogenous EVs. However, pHluorin has shortcomings, including low expression level in target cells and fast photobleaching. In addition, the small penetration depth of the fluorescent signal further limits the application in clinical translation.

Apart from fluorescent proteins with changeable signals upon environment change, gene editing tools can also change the fluorescent signal following activation. The Cre-loxP technology allowed gene ablation in cells, and thus it was applied to monitor EV transfer between highly metastatic human MDA-MB-231 tumor cells and less malignant human T47D tumor cells in mouse in real time (Zomer et al., 2015; Zomer et al., 2016). For this purpose, MDA-MB-231 cells were engineered to express the Cre recombinase and CFP, while T47D cells were edited to express floxed-DsRed-floxed-eGFP. When T47D cells internalized EVs from MDA-MB-231 cells, their fluorescent signals turned from red to green. High-resolution intravital imaging observed that EVs derived from malignant cells were internalized by less malignant tumors, both located near and distant places. However, the efficiency of tracking might be low, as only mRNAs of Cre recombinase but not the proteins were detected in EVs derived from the engineered donor cells in this study. The CRISPR-Cas9 system is another popular gene editing tool in molecular and cellular biology (Wang et al., 2016). Ye *et al.* employed this strategy for EV tracking in cells and *in vivo* (Ye et al., 2020; Ye et al., 2022). The author loaded the single-guide RNA, Cas9 ribonucleoprotein complex, into EVs by editing the donor cells to express the complex tagged with GFP nanobody and CD63-GFP at the same time, resulting in a high loading efficiency. Then, the genetically engineered melanoma cells were implanted subcutaneously in a mouse model that ubiquitously expressed STOP-tdTomato. By analyzing the tissue sections, they found that EVs from the tumor xenografts preferentially targeted the brain and liver as indicated by the fluorescent signal of tdTomato. The method for cargo loading in this study was efficient, and the *in vivo* tracking was noninvasive and real-time, making it a powerful method.

Apart from the aforementioned tools, monitoring EVs by the changeable fluorescence signal could be toggled by a fine design. He et al. (2019) developed a novel device to track the EV internalization pathway by using the ATP aptamer and its complementary sequence. The unit included the ATP aptamer with Cy3 modification at the 3’ end, its complementary sequence with a BHQ-1 dye at the 5’ end, and a FAM dye at the 3’ end. The whole unit was anchored on EVs by lipid insertion. Recipient cell membranes exhibited green in case of membrane fusion, and its cytosol demonstrated red in case of endocytosis. Thus, the EV uptake pathway by distinct cells was clearly visible by fluorescent imaging. This method was efficient on monitoring the EV uptake pathway and possessed great potential for EV tracking *in vivo*.

### 4.2 Bioluminescent imaging

Bioluminescent imaging is the most widely used *in vivo* imaging technique (Yi et al., 2020). The bioluminescence has advantages of high sensitivity, low background, and credible results, as the labeling is intrinsic. However, it requires genetic modification and substrate injections. Moreover, bioluminescence tracking is limited by the facts that the signal fades quickly and the penetration ability and spatial resolution are low. Representative examples are listed in Table 2.

Several kinds of luciferases were employed for EV labeling and tracking. Gangadaran et al. (2017) employed the Renilla luciferase (RLuc) for EV tracking *in vivo* by transducing RLuc into the donor cells. Then, EVs derived from different cells having different bio-distribution patterns were revealed by *in vivo* bioluminescence imaging. EVs isolated from thyroid cancer cells emitted strong signals at the lungs followed by the liver, spleen, and kidney, while EVs derived from breast cancer cells showed strong signals at the liver, followed by the lung, spleen, and kidney. In this study, luciferases were introduced into EVs by merely overexpressing RLuc in donor cells without any EV-guiding module. Thus, it was very likely that the loading efficiency was unsatisfactory, and free luciferases might be released in the cell culture media and be co-isolated with EVs by ultracentrifugation, leading to fake signals.

Compared with the regular luciferases, including Gaussia luciferase (GLuc) and RLuc, NanoLuc has a larger dynamic range and high signal intensity, making it more suitable for *in vivo* tracking (Gupta et al., 2020). Gupta et al. (2020) performed the *in vivo* tracking of EVs with NanoLuc, which was genetically ligated to CD63 to improve the loading efficiency. Nevertheless, free NanoLuc was detected in the cell culture medium. For this reason, they examined the tracking results of other luciferases and identified ThermoLuc as the best luciferase for the *in vivo* imaging of EVs (Gupta et al., 2020). Tracking with ThermoLuc indicated that EV bio-distribution was different according to different injection routes. Moreover, they tracked EVs from different subtypes by tagging ThermoLuc to CD63 or CD9 and concluded that different subtypes had different bio-distribution patterns.

### 4.3 Nuclear imaging

Nuclear imaging is broadly used in cellular biology and clinical diagnosis. The labeling is durable, and the imaging is highly sensitive and has high penetrability. However, it is expensive, time-consuming, and unavailable for most laboratories. Representative examples are listed in Table 2.

For radiolabeling, the radioactive substances are easily introduced into EVs by incubation. The *in vivo* imaging was then obtained by positron emission tomography (PET) or single-photon emission computed tomography (SPECT) (Khan, 2021). Gangadaran et al. (2018) produced ^99m^Tc-labeled nanovesicles by extruding the red blood cells, and the *in vivo* imaging indicated that the nanovesicles were highly distributed in the liver and spleen but not in the thyroid. Molavipordanjani et al. (2020) also labeled EVs with ^99m^Tc and found that tumor cells derived EVs, which were genetically modified to have a ligand of HER2 on the membrane surface, showed a high accumulation in xenograft tumor generated with high-HER2-expression tumor cells.

Radioactive substances can also be engineered on the EV surface by chemical ligation. Faruqu et al. (2019) compared the ^111^indium labeling efficiency of intraluminal labeling by incubation and membrane labeling by chemical ligation and concluded that membrane-labeled melanoma cell-derived EVs had a superior radiolabeling efficiency and radiochemistry stability. Then, the *in vivo* distribution of the membrane-labeled EVs was imaged by the SPECT scan in melanoma-bearing mice. The labeled EVs accumulated primarily in the liver and spleen, followed by the kidneys. Similarly, Banerjee et al. (2019) labeled small EVs with ^64^Cu by decorating the metal chelator, 1,4,7,10-tetraazacyclododecane-1,4,7,10-tetraacetic acid (DOTA), on the membrane surface *via* thiol–maleimide conjugation. By magnetic resonance imaging (MRI) and PET, they observed the accumulation of small EVs derived from human umbilical cord blood mononuclear cells (hUCB-MNCs) in mice at 1 h, 1.5 h, 2 h, and 3 h after intravenous injection.

### 4.4 Nanoparticle-assisted imaging

Nanoparticles have been extensively employed for biomedical imaging and have great potential as probes for disease diagnosis. Among them, gold nanoparticles (GNPs), quantum dots (QDs), and metal–organic framework (MOF) are powerful tools for EV tracking. Representative examples are listed in Table 2.

#### 4.4.1 Gold nanoparticles (GNPs)

GNPs can be used for cellular biomedical imaging due to their unique plasmonic properties. Other than the optical modalities, the signal of GNPs can penetrate deep inside the tissues, which benefits the imaging of the deep body structure. Betzer et al. (2017) employed glucose-coated GNPs for tracking EVs in the deep brain structure. EVs were labeled through active uptake of glucose-coated GNPs and *in vivo* images were taken by computed tomography, indicating that the intranasal administration resulted in the superior brain accumulation of EVs compared with intravenous injection. They further noninvasively tracked the intranasally administered EVs in the mouse model with focal brain ischemia and found that EVs had an increased accumulation at the focal site over 24 h.

#### 4.4.2 Quantum dots (QDs)

QDs are nanoscale crystals that can emit fluorescence of various wavelengths upon being excited by UV light. Compared with the conventional fluorophore, QDs have brighter emission, a higher signal-to-noise ratio, and less photobleaching, making them attractive for imaging. Chen et al. (2015) employed QDs for noninvasively tracking MVs *in vivo*. MVs were labeled with QDs *via* binding between biotin and streptavidin. Then, with a mouse model bearing melanoma xenografts, they found that MVs derived from malignant melanoma cells administered intratumorally could target tumor cells but not transfer to the liver, in which injected-free QDs were distributed.

#### 4.4.3 Metal–organic framework (MOF)

MOFs, composed of metal ions and the coordinated organic ligands, have attracted extensive interests for biomedical research, such as biological imaging and delivery. Illes et al. (2017) coated the iron-based MOFs, MIL-88A NPs, with EVs by the fusion method. In this study, they loaded calcein to the MOFs for imaging. In addition, the iron-based MOFs are capable of MRI imaging, so it is worth expecting in their future work.

### 4.5 Other tracing methods

#### 4.5.1 Photoacoustic imaging

Photoacoustic imaging (PAI), developed based on the photoacoustic effect, is an attractive imaging modality as it allows noninvasive and real-time imaging with the advantages of deep tissue penetration and high spatial resolution. Proper contrast agents are needed for PAI, which absorb the laser irradiation energy and convert it into the ultrasound signal. Apart from the contrast agents, PAI is similar to ultrasound imaging, which is cheaper than CT and MRI, and has no risk of radiation exposure. Moreover, the contrast agents, which could emit optical signals, allow optical imaging with the advantage of high contrast at the same time. Representative examples are listed in Table 2.

PAI is employed for EV imaging in several studies. Ding et al. (2019) encapsulated the H_2_O_2_-sensitive PAI contrast agent into the erythrocyte membrane by extrusion. The generated nanoparticles could image the nasopharyngeal carcinoma cells, which produces H_2_O_2_ following laser radiation. The strategy combines the excellent biocompatibility and stability of the encapsulated nanoparticles, the sensitive and specific reactions between the nanoparticles and cancer cells, and the high spatial resolution of PAI, present as an ideal platform for tumor diagnosis. In addition to the application in diagnosis, Jang et al. (2020) developed the photodynamic and immune therapy assisted by PAI. The photosensitizer, chlorin e6, was loaded into the isolated tumor cell-derived EVs by sonication. The generated nanoparticles could be visualized *via* PAI *in vivo* and produce cytotoxic reactive oxygen species (ROS) inside the tumor cells under the tissue-specific laser irradiation. Tumor cells were damaged by tumor cell-derived EV-stimulated ROS and cytokines from immune cells.

#### 4.5.2 Raman spectrum for imaging

Raman spectroscopy is an analytical technique developed based on the inelastic scattering of the molecular vibration and widely applied in many research fields, as it can reveal the fingerprint spectrum of the samples in a non-destructive and label-free manner. Raman spectroscopy is applied to analyze the compositional characterization of purified EVs. EVs from different subtypes and different cells could be distinguished by Raman spectroscopy, and the latter had great potential in developing diagnostic approaches for early-stage cancer (Park et al., 2017).

However, to trace EVs in cells or in tissues, signal of EVs can be confounded by the cells’ signal as the similar chemical bond they possess. The introduction of Raman tags, such as alkyne and carbon–deuterium bonds, which vibrates in the region different from the endogenous biological molecules, can circumvent this issue. Horgan et al. (2020) introduced the carbon–deuterium bonds to EVs by metabolic incorporation. Then, by using the confocal spontaneous Raman microspectroscopy system, they obtained a high-resolution image of EV uptake by cells *in vitro*, both in 2D and 3D.

#### 4.5.3 Quantitative analysis of EVs distributed *in vivo* with YRNA-based amplification

In addition to the involvement of different imaging modalities, the quantitative measurements of EVs distributed in different tissues can be obtained by nucleic acid-based amplification. YRNAs, first discovered as the RNA component of Ro RNA particle in 1981 and having a direct role in DNA replication, are particularly plentiful in EVs (Kowalski and Krude, 2015). Among all the YRNAs, NT4, a 24-nuleotide 5’ fragment of human YRNA 4, was highly enriched in human CDC-derived EVs but absent in mouse, which could act as the tracer of EVs in mouse (Ciullo et al., 2022). Then, by using the quantitative PCR approach, the distribution of CDC-derived EVs in the mouse and mouse cells was measured.

#### 4.5.4 Multimodal imaging

Multimodal imaging refers to the production of two or more sets of signals at the same time, such as the combination of optical and magnetic imaging. It is attractive as it can provide multiplex information for one sample, especially when the involved modalities have complementary advantages. Several studies tracked EVs by multimodal imaging. Zhao et al. (2016) encapsulated Mn-magnetofunctionalized Ag_2_Se QDs (Ag_2_Se@Mn QDs) in MVs by sonication, which had great near-infrared fluorescence and magnetic resonance imaging capabilities. Further, they carried out the continuous dual-modal tracking of MVs in nude mice after the intraperitoneal injection and realized the long-term, noninvasive, whole-body, high-resolution, *in situ* quantitative, and dual-mode tracking of MVs *in vivo*.

## 5 Conclusion

Developments in the field of EVs offer very promising approaches for the diagnosis and treatment of diseases. However, the current understanding of these EVs, especially their *in vivo* behavior and distribution, is still insufficient. Current methods for *in vivo* imaging and tracking of EVs, including fluorescent imaging, bioluminescent imaging, photoacoustic imaging, nuclear imaging, and MRI have greatly aided our understanding of the uptake mechanism of EVs, bio-distribution patterns, migration, and functions of EVs and more importantly, pharmacokinetic properties can be assessed and dosage can be optimized by these tracking methods. The development of a reliable, non-invasive, and stable EV labeling technology and *in vivo* imaging technology to reveal their EVs in disease diagnosis, drug delivery, and barrier penetration will promote further clinical applications of EVs.

Tracking EVs in cells or *in vivo* provides information involved in cellular uptake and cell–cell communication. Various uptake mechanisms of EVs have been reported. He et al. (2019) visualized different uptake pathways using a fine-designed fluorescent device. The results indicated that the occurrence of uptake pathways was different with different EV origins and recipient cell types. Specific cell–cell communication *in vivo* could be monitored by EV tracking as well. Lai et al. (2015) visualized the communication between tumor cells and surrounding cells *via* EV exchange through fluorescent labeling. Also, Wiklander et al. (2015) reported that EV *in vivo* bio-distribution was determined by cell source, route of administration, and targeting. In addition to the mechanism study, EV tracking has an instructive function for laboratorial and clinical research studies. Betzer et al. (2017) found that intranasally administered EVs had enhanced brain accumulation compared to intravenously injected EVs.

Despite promising applications, several important aspects remain to be explored in future research studies. First, the labeling of EVs with fluorescent dyes or genetic engineering techniques is limited by the inherent background generated by natural biomolecules such as hemoglobin and lipoproteins, which can significantly interfere with the staining process of EVs and lipophilic dyes, resulting in high contamination during sample isolation, thereby affecting the *in vivo* distribution. The evaluation of *in vivo* administration routes based on optical imaging is limited to scientific research, while the conversion of optical imaging to humans is largely hindered by the limited penetration of light. Also, EVs lack standard procedures. For example, the number of injected EVs or the labeling efficiency of EVs is not uniform in various literatures. Therefore, dose normalization is recommended for future studies in order to obtain comparisons between studies. Currently, there is almost a lack of imaging platforms dedicated to exosomal dynamic tracking. Due to the lack of an EV-specific imaging platform system, it is necessary to perform multimodal imaging methods to reveal the true fate of EVs, such as using MRI and radionuclide imaging platforms (PET or SPECT) to improve its high sensitivity. Moreover, these multimodal imaging instruments are readily available in the clinic and can be easily extended from research to clinics. It is believed that elucidating the unclear behavior and function of EVs *in vivo* may facilitate its translation into clinical applications. As tagging methods and tracking techniques improve, the mysteries of EVs will be unraveled.
